# The Effects of Mixed Inoculum Storage Time on In Vitro Rumen Fermentation Characteristics, Microbial Diversity, and Community Composition

**DOI:** 10.3390/ani15010005

**Published:** 2024-12-24

**Authors:** Chang Liu, Jing Ge, Jiaqi Dai, Mingren Qu, Kehui Ouyang, Qinghua Qiu

**Affiliations:** Jiangxi Province Key Laboratory of Animal Nutrition and Feed, College of Animal Science and Technology, Jiangxi Agricultural University, Nanchang 330045, China

**Keywords:** bacterial community composition, bacterial diversity, in vitro fermentation, mixed inoculum, rumen fermentation characteristic, storage time

## Abstract

This study aimed to investigate the effects of different storage times of the mixed inoculum on in vitro rumen fermentation characteristics, microbial diversity, and community composition. The experiment was divided into five groups, with mixed inoculum composed of fresh rumen fluid and culture medium being stored at 39 °C for 0 h (H0), 12 h (H12), 24 h (H24), 36 h (H36), and 48 h (H48). After 48 h of in vitro fermentation, the fermentation fluid was collected to assess rumen fermentation characteristics and microbial community composition. The H24 group showed higher total gas production, ammoniacal nitrogen levels, and total volatile fatty acids, as well as higher concentrations of individual volatile fatty acids except propionate, compared to the H0 and H48 groups (*p* < 0.05). The Shannon and Simpson evenness indices were significantly higher in the H0, H12, and H24 groups than in the H48 group (*p* < 0.05). A total of nine phyla and sixteen genera involved in starch and fiber degradation were found to be more abundant in the H24 or H48 groups (*p* < 0.05). Moreover, nine predicted metabolic pathways were observed to be significantly enriched in either the H24 or H48 group (*p* < 0.05). Both principal coordinates analysis (PCoA) and non-metric multidimensional scaling (NMDS) analysis revealed distinct clustering patterns among the H0, H12, H24, H36, and H48 groups, and analysis of similarities (ANOSIM) confirmed these significant differences (R = 1.00, *p* < 0.05). This study demonstrates that the storage time of mixed inoculum influences rumen fermentation characteristics and microbial community composition in a time-dependent manner. It is recommended to use a mixed inoculum that has been stored within 24 h in an anaerobic environment at 39 °C for in vitro rumen fermentation tests. This study offers valuable microbial insights into the storage strategies for mixed inoculum, thereby improving the methodologies for variable control in in vitro rumen fermentation techniques.

## Simple Summary

In vitro rumen fermentation is a key technique for evaluating the nutritional value of feeds or feedstuff for ruminants, yet its accuracy is heavily dependent on the rumen fluid used. To minimize discrepancies arising from varying rumen fluids, enhancing preservation techniques to maintain consistent rumen fluid quality is a promising strategy. While much of the existing research has focused on preserving rumen fluid, the actual application of this technique involves a mixed inoculum, which is a combination of rumen fluid and artificial culture medium. This study aimed to investigate how different storage times of the mixed inoculum affect in vitro rumen fermentation characteristics and microbial community composition. Our findings revealed variations between the mixed inoculum stored at the 0 h mark and those stored for 12, 24, 36, and 48 h, with particularly notable differences at the 24 h and 48 h marks. Based on these results, it is advised to use a mixed inoculum stored within 24 h for in vitro rumen fermentation tests. This study monitored the dynamic changes in rumen fermentation characteristics and microbial communities across various storage times of the mixed inoculum, providing valuable insights for refining the control of variables in in vitro rumen fermentation techniques.

## 1. Introduction

Rumen fluid is a complex mixture composed of water, electrolytes, vitamins, and a diverse array of microorganisms essential for the digestion of fibrous plant material in ruminants [1]. It is characterized by its rich microbial population, which includes bacteria, protozoa, fungi, and archaea. These microorganisms are responsible for breaking down cellulose and hemicellulose into volatile fatty acids (VFA) that serve as the primary energy source for the host animal [2]. The composition of rumen fluid is dynamic and is influenced by factors, including diet, climate, and the health status of the animal [3]. Understanding the composition of rumen fluid is essential for optimizing animal nutrition and health, as it directly affects feed efficiency and overall productivity [4].

Rumen fluid serves as an excellent medium for in vitro fermentation cultures due to its complex microbial composition and enzymatic activities, which closely mimic the natural digestive processes in ruminants [5]. The use of rumen fluid in vitro offers a valuable tool for studying the effects of different substrates on microbial activity and for evaluating the potential of novel feed additives [6]. To ensure the reliability of results, in vitro rumen fermentation often requires multiple batches [7]. Nonetheless, variations in rumen fluid, whether due to differences among individuals or the timing of collection, can significantly affect the final assessment outcomes [8]. To address this issue, using the same rumen fluid across all batches in in vitro fermentation trials may provide an effective solution.

Many studies have been conducted to investigate how different storage conditions of rumen fluid affect in vitro fermentation outcomes. Maintaining rumen fluid under proper temperature conditions, such as refrigeration at 4 °C or on crushed ice, freezing at −20 °C or −80 °C, or preservation in liquid nitrogen, has proven to be an effective preservation technique [9,10,11]. This ensures the rumen fluid’s viability as an inoculum for subsequent in vitro fermentation assays. Several studies have indicated that maintaining rumen fluid at 40 °C for 5 h or at 18 °C for 48 h did not significantly affect the degradation characteristics of feed during in vitro fermentation assessments [12,13]. Beyond the previously mentioned strategies, using cryoprotectants like glycerol and dimethyl sulfoxide to rumen fluid during preservation has been shown to enhance gas and VFA production in in vitro fermentation tests [10,14]. Zhao et al. [15] found that rumen fluid stored at −20 °C with dimethyl sulfoxide for 180 days showed no difference in methane and VFA production compared to fresh rumen fluid.

Recent studies have also focused on the effects of storage conditions of rumen fluid on its physicochemical properties and microbial communities. Previous reports have found that rumen fluid stored at 4 °C for 96 h exhibited similar pH values and VFA concentrations to fresh rumen fluid [16]. Martin et al. [17] also reported comparable findings, noting that rumen fluid stored at 38 °C for up to 9 h or at ambient temperature for 2 h maintained properties and viabilities akin to those of fresh rumen fluid. Qiu et al. [18] revealed that the majority of fermentation characteristics in rumen fluid were significantly altered after a 60-day preservation period and that changes in the rumen bacterial community profile were observed within 30 days of preservation. Takizawa et al. [19] discovered that rumen fluid, when stored at 4 °C for up to 7 days, maintained high levels of fibrolytic activity and served as a suitable source of organic carbon for methane fermentation of wastepaper. In a longitudinal study spanning 240 days, it was found that there were no differences in rumen fermentation characteristics, microbial diversity, or community composition between rumen fluid stored at −20 °C and −80 °C [18].

The in vitro fermentation technique allows researchers to investigate the fermentation dynamics and metabolic activities of rumen microbes under controlled conditions. The mixed inoculum used in this technique is typically prepared by mixing rumen fluid with a culture medium in a 1:2 volume ratio [7]. However, preparing the culture medium is not only labor-intensive and time-consuming but also prone to variations between batches [7]. If the intrinsic characteristics and incubation outcomes of the mixed inoculum remain largely consistent over a short interval, it is feasible to proceed directly with subsequent in vitro batch trials using the same mixed inoculum. This strategy not only streamlines the experimental process but also effectively reduces the variability introduced by fluctuations in both rumen fluid and culture medium. While previous studies have focused on investigations using single rumen fluid, no reports have examined the effect of storage time for the mixed inoculum on in vitro rumen fermentation. Therefore, the aim of this experiment is (1) to investigate the dynamic changes in in vitro fermentation characteristics and rumen microbes under different storage times of the mixed inoculum, and (2) to provide recommendations for feasible storage times for the mixed inoculum. It is hypothesized that short-term storage (less than 24 h) of the mixed inoculum will not affect fermentation characteristics and rumen microbes, while long-term storage (beyond 24 h) is expected to result in significant changes.

## 2. Materials and Methods

### 2.1. Animal Ethics

This research was conducted with strict compliance with the animal care and welfare guidelines. The protocols were reviewed and approved by the Committee for the Care and Use of Experimental Animals at Jiangxi Agricultural University. The protocol number is JXAULL-20220306.

### 2.2. Rumen Fluid Acquisition, Inoculum Preparation, and Experimental Design

Rumen fluid was collected from five dairy cows fed a diet composed of corn silage (23.87%), alfalfa hay (18.56%), oat hay (2.64%), corn (25.69%), soybean meal (3.01%), wheat (7.65%), wheat bran (2.67%), beet pulp (1.81%), molasses (2.54%), cottonseed (8.53%), fat-energy powder (1.29%), dicalcium phosphate (0.62%), salt (0.57%), and premix (0.55%). This diet provided the following nutrients: crude protein (CP) at 16.90%, net energy for lactation (NEL) at 1.75 Mcal/kg, neutral detergent fiber (NDF) at 31.01%, acid detergent fiber (ADF) at 23.10%, crude fat at 5.56%, calcium at 0.85%, and phosphorus at 0.42%. The rumen contents, comprising both liquid and solid phases, were collected one hour before morning feeding using a rumen fistula technique, as described by Paz et al. [20]. These contents were then filtered through a four-layer cheesecloth to obtain the rumen fluid. The rumen fluids from the five cows were pooled and homogenized to create a uniform rumen fluid mixture, which was finished within 10 min and then served as the rumen fluid component of the inoculum.

The cultivation medium was prepared with the following components: 47.56% distilled water, 0.01% trace element solution, 23.78% artificial saliva, 23.78% constant element solution, 0.1% resazurin solution, and 4.76% reducing agent solution, and the precise formulation of each solution is described in the study by Zheng et al. [21]. The rumen fluid was thoroughly mixed with cultivation medium at a volume ratio of 1:2 to create the mixed inoculum, and this operation was completed within 10 min. This mixed inoculum was then transferred into a sealed container filled with carbon dioxide and incubated in a water bath at a constant 39 °C. The fermentation characteristics of the mixed inoculum under different storage times are listed in Table 1.

The mixed inoculum was collected at 0, 12, 24, 36, and 48 h post-incubation, specifically for subsequent in vitro fermentation tests. At the 0 h time point, four culture bottles were used, and for each subsequent time point, five culture bottles were used to ensure experimental replication. Additionally, three blanks were designed to correct gas production from the substrate. The fermentation results obtained from the mixed inoculum at storage time points of 0 h, 12 h, 24 h, 36 h, and 48 h were labeled H0, H12, H24, H36, and H48, respectively.

### 2.3. In Vitro Fermentation Test

For the in vitro fermentation tests, the substrate used was identical to the diet of the rumen fluid donor cows mentioned above. Specifically, 0.40 g of the substrate was precisely weighed and placed into a 125 mL culture bottle. Then, 60 mL of mixed inoculum from different storage times was injected into the culture bottle. The oxygen in the bottle was removed by flushing it with carbon dioxide, and the bottle was incubated in a thermostatic water bath (SHA-B, Changzhou Guohua Electric Appliance Co. Ltd., Changzhou, China) for 48 h, maintaining a consistent water temperature of 39 °C and shaking speed of 50 r/min. During this incubation, the gas production was recorded every three hours using a graduated syringe. Ice blocks were applied to terminate the fermentation process, and the measurement of the pH value of the fermentation liquid was performed without delay. The fermentation liquid was immediately transferred to a −80 °C freezer for further analysis of rumen fermentation characteristics and microbiome sequencing.

### 2.4. Parameter Measurement

The pH value was swiftly ascertained upon acquisition of the fermented fluid, utilizing a portable pH meter (Testo 206, Testo AG, Schwarzwald, Germany). The ammoniacal nitrogen (NH_3_-N) concentration was assessed via the phenol–hypochlorite reaction method, adhering to the protocol established by Broderick and Kang [22]. The microbial protein (MCP) concentration was determined using the improved Lowry’s assay method [23]. The VFA detected in this research included acetate, propionate, isobutyrate, butyrate, isovalerate, and valerate. The branched-chain volatile fatty acids (BCVFA) included isobutyrate, isovalerate, and valerate. The individual VFA were quantified using a gas chromatograph (GC-2014 Shimadzu Corporation, Kyoto, Japan), following the procedures described in Qiu et al. [24].

### 2.5. DNA Extraction, Sequencing, and Data Analysis

A total of 24 fermented liquid samples, 4 from the H0 group and 20 from the H12, H24, H36, and H48 groups, were selected for DNA extraction using a DNA bacterial extraction kit (OMEGA, Omega Bio-tek, Inc., Norcross, GA, USA). The extraction process followed the manufacturer’s instructions strictly. The Nanodrop 2000 spectrophotometer (Thermo Fisher Scientific, Inc., Waltham, MA, USA) was used to assess the purity and concentration of the extracted DNA. After assessment, all 24 DNA samples were found to be of high quality, suitable for further processing. Universal primers 338F (5′-ACTCCTACGGGAGGCAGCAG-3′) and 806R (5′-GGACTACHVGGGTWTCTAAT-3′) were used to amplify the V3–V4 region of the bacterial 16S rRNA gene. An 8 bp barcode sequence was added to the 5′ end of both the forward and reverse primers for sample differentiation. The amplification, incorporating barcode sequences, was performed on the ABI 9700 PCR instrument (Applied Biosystems, Inc., Waltham, MA, USA), following the PCR amplification system and reaction program detailed by Qiu et al. [25]. Following amplification, a 1% agarose gel electrophoresis was used to verify the size of the target bands. The PCR products were then purified using the Agencourt AMPure XP (Beckman Coulter, Inc., Brea, CA, USA) nucleic acid purification kit. Library construction was carried out on the purified PCR products using the NEB Next Ultra II DNA Library Prep Kit (New England Biolabs, Inc., Ipswich, MA, USA). The libraries were further purified using the Agencourt AMPure XP (Beckman Coulter, Inc., Brea, CA, USA) nucleic acid purification kit. The Agilent 2100 Bioanalyzer (Agilent Technologies, Inc., Santa Clara, CA, USA) was used to determine the library fragment sizes, and the ABI StepOnePlus Real-Time PCR System (Applied Biosystems, Inc., Waltham, MA, USA) was utilized for the precise quantification of library concentrations. Finally, the libraries were sequenced on the Nextseq 2000 platform (Illumina, Inc., San Diego, CA, USA) using a PE300 sequencing strategy. The raw sequencing data were deposited in the NCBI’s Sequence Read Archive (SRA) database with the accession number PRJNA1185238.

The raw sequencing data were partitioned into distinct samples according to their barcode sequences. The Paired-End reAd mergeR (PEAR, version 0.9.6) software was used for the filtration and assembly of the reads, eliminating sequences that contained the ambiguous nucleotide ‘N’ and discarding any regions with quality scores falling below 20. The assembly process was configured with a minimum overlap of 10 bp and a *p*-value threshold of 0.0001. Following assembly, Vsearch (version 2.7.1) was applied to filter out sequences that were either shorter than 260 bp or exceeded 500 bp in length. Additionally, chimera detection and removal were performed by aligning sequences against the Gold Database, employing the UCHIME method. Usearch (version 10.0.240) with the Unoise algorithm was used to refine the dataset and eliminate noise, identifying Amplicon Sequence Variants (ASVs). To maintain uniform coverage across all samples, the sequencing depth for all samples was standardized to 58,256 reads. The ASVs were then aligned against the SILVA138 database with an e-value cutoff of 1 × 10^−5^ to ascertain the taxonomic affiliations for each variant. Subsequently, alpha diversity indices, including Chao 1, observed species, phylogenetic diversity (PD) whole tree, Shannon index, and Simpson index, were computed for each sample using QIIME 2, based on the ASVs and their respective abundances. Principal coordinates analysis (PCoA) and non-metric multidimensional scaling (NMDS) were utilized to reveal the distinctions among different storage times based on Bray–Curtis dissimilarities. Additionally, analysis of similarities (ANOSIM) was performed to assess the similarities within the H0, H12, H24, H36, and H48 groups, employing the vegan packages in R software (version 4.3.1, R Foundation for Statistical Computing, Vienna, Austria). Linear Discriminant Analysis Effect Size (LEfSe) was applied to identify biomarkers that exhibited statistically significant differences, using Python (version 3.10) with an LDA score threshold set at 4. Furthermore, Phylogenetic Investigation of Communities by Reconstruction of Unobserved States (PICRUSt) was employed to predict the metagenomic functions of the detected microbial communities, thereby shedding light on the inherent ruminal functions associated with the bacterial microbiota.

### 2.6. Statistical Analysis

Data in this study were analyzed using the MIXED procedure of SPSS (version 20, IBM, Chicago, IL, USA) with the following model: Y*_ij_* = μ + T*_i_* + R*_j_* + e*_ij_*, where Y*_ij_* represents the dependent variable, μ is the overall mean, T*_i_* denotes the fixed effect of mixed inoculum storage time (*i* = 0, 12, 24, 36, 48), R*_j_* signifies the random effect of the bottle, and e*_ij_* is the residual effect. The results are presented as the averages and standard error of the mean (SEM). Post hoc comparisons among storage times of mixed inoculum were conducted using Tukey’s tests. A significant difference was considered when *p* < 0.05.

## 3. Results

### 3.1. Total Gas Production

**Figure 1:**
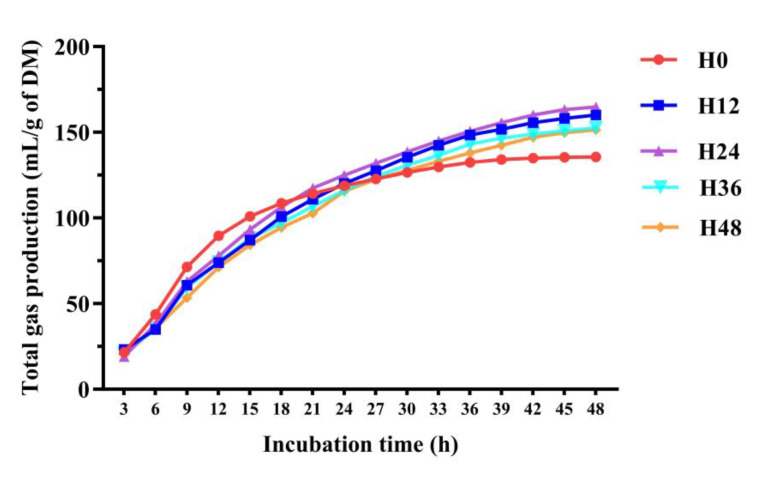
Gas production dynamics in response to incubation time across various mixed inoculum storage times. H0, H12, H24, H36, and H48 indicate the mixed inoculum used in the in vitro fermentation tests was stored for 0 h, 12 h, 24 h, 36 h, and 48 h, respectively.

The variations in total gas production across various storage times of the mixed inoculum are depicted in Figure 1. There was a notable surge in gas production across all groups prior to 36 h of incubation. After 42 h, gas production plateaued, suggesting that the 48 h incubation period used in this study is sufficient to capture the fermentation dynamics under the given substrate conditions. The H24 group showed greater total gas production compared to the H0 group (*p* < 0.05), but it did not exhibit significant differences when compared with the H12, H36, and H48 groups (Table 2).

### 3.2. Rumen Fermentation Characteristics

The effects of mixed inoculum storage time on in vitro rumen fermentation characteristics are detailed in Table 2. The H48 group exhibited a higher pH value than the H24 group (*p* < 0.05), and the concentration of NH_3_-N was inversely related to this. The H0 group had a higher concentration of MCP than the H12, H24, H36, and H48 groups, yet its total VFA content was lower than that of the H12, H24, and H36 groups (*p* < 0.05). Regarding individual VFA concentrations, all except propionate varied across different storage times, with the H0 group consistently showing lower levels than the H12, H24, and H36 groups (*p* < 0.05). The proportions of individual VFA mirrored these concentration trends. Notably, the H0 group had a higher proportion of propionate than the other groups (*p* < 0.05).

### 3.3. Rumen Bacterial Alpha Diversity

The effects of mixed inoculum storage time on alpha diversity metrics of the rumen bacterial community are presented in Table 3. Richness, as measured by the Chao1 and observed species indices, was not affected by storage time (*p* > 0.05), but evenness was significantly impacted. Specifically, the PD whole tree for the H12 group was significantly higher than those for the H48 group, and the Simpson index and Shannon index for the H0, H12, and H24 groups were significantly higher than those for the H48 group (*p* < 0.05).

### 3.4. Rumen Bacterial Community Composition

The effects of mixed inoculum storage time on the structure of rumen bacterial communities at the phylum and genus levels are documented in Table 4 and Table 5, respectively. At the phylum level, Bacteroidota and Firmicutes were the most prevalent. Bacteroidota showed a significantly higher relative abundance in the H12, H24, H36, and H48 groups compared to the H0 group (*p* < 0.05). In contrast, Firmicutes had a significantly higher relative abundance in the H0 group (*p* < 0.05). The H0 group also had a significantly higher relative abundance of Proteobacteria compared to the H24 group (*p* < 0.05), while Verrucomicrobiota showed an inverse pattern (*p* < 0.05). The relative abundance of Euryarchaeota in the H12 group was significantly higher than in the H24, H36, and H48 groups (*p* < 0.05). Patescibacteria in the H24 group had a significantly greater presence than in the H0, H36, and H48 groups (*p* < 0.05). Desulfobacterota in the H36 group exhibited a significantly higher relative abundance than in the H0 group (*p* < 0.05). Additionally, the relative abundances of Fusobacteriota and Spirochaetota in the H48 group were significantly higher than in the H0, H12, and H24 groups (*p* < 0.05).

At the genus level, 14 genera exhibited an average relative abundance exceeding 1%, with 13 displaying significant differences across the groups. Specifically, the H0 group showed significantly reduced relative abundances of Rikenellaceae RC9 gut group, Probable genus 10, Lachnospiraceae FCS020 group, and Lachnospiraceae AC2044 group when compared to the H36 and H48 groups (*p* < 0.05). Conversely, the relative abundances of Succiniclasticum, Succinivibrio, Succinivibrionaceae UCG-002, Pseudobutyrivibrio, and Butyrivibrio were significantly higher in the H0 group relative to the H36 and H48 groups (*p* < 0.05). Furthermore, Succiniclasticum showed a higher relative abundance in the H0, H12, and H24 groups compared to the H36 and H48 groups, while Ruminobacter, Anaerovibrio, and Bacillus are significantly more abundant in the H48 group than in the H12 group (*p* < 0.05).

### 3.5. Rumen Bacterial Beta Diversity

**Figure 2:**
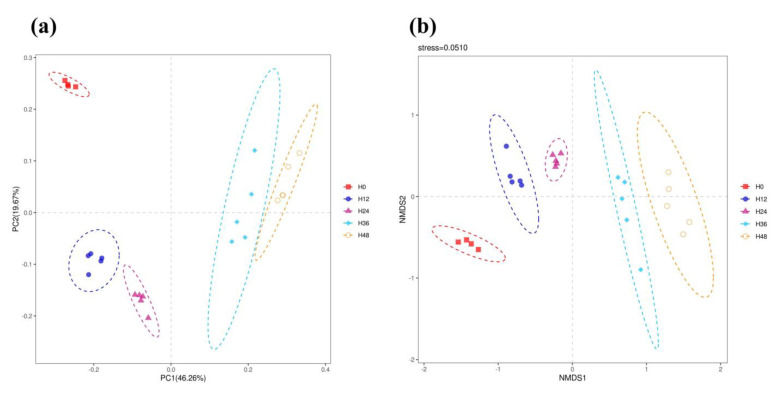
Rumen bacterial beta diversity across different storage times of mixed inoculum. (**a**) Principal coordinates analysis (PCoA); (**b**) non-metric multidimensional scaling (NMDS). H0, H12, H24, H36, and H48 indicate the mixed inoculum used in the in vitro fermentation tests was stored for 0 h, 12 h, 24 h, 36 h, and 48 h, respectively.

As illustrated in Figure 2, both PCoA and NMDS revealed distinct clustering patterns among the H0, H12, H24, H36, and H48 groups. The ANOSIM analysis further substantiated these findings, indicating significant disparities in the rumen bacterial community composition when comparing the H0 group with the subsequent groups: H12 (R = 1.00, *p =* 0.006), H24 (R = 1.00, *p =* 0.007), H36 (R = 1.00, *p =* 0.004), and H48 (R = 1.00, *p =* 0.010). These results collectively highlight the substantial influence that the mixed inoculum storage time has on the rumen bacterial community structure.

### 3.6. Biomarker Analysis

**Figure 3:**
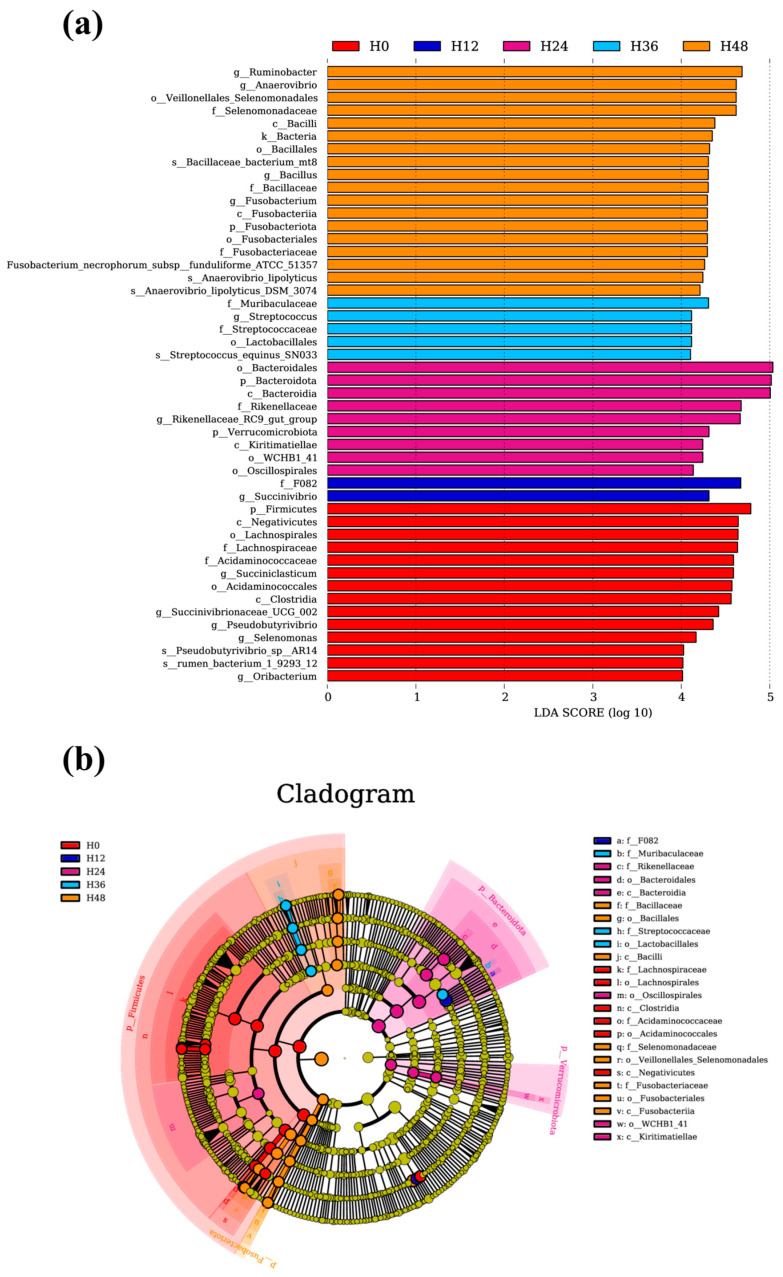
Illustration of the effects of mixed inoculum storage times on discriminative bacterial communities at multiple taxonomic levels: (**a**) linear discriminant analysis; and (**b**) cladogram. H0, H12, H24, H36, and H48 indicate the mixed inoculum used in the in vitro fermentation tests was stored for 0 h, 12 h, 24 h, 36 h, and 48 h, respectively.

Biomarker analysis could reveal a more comprehensive set of differential microbes across various taxonomic levels. In addition to the aforementioned differential microbes and their affiliations, 17 additional microbes have been identified, as shown in Figure 3. These differential microbes were c_Negativicutes, f Acidaminococcaceae, o_Acidaminococcales, c_Clostridia, g_Selenomonas, and g_Oribacterium in the H0 group; f_F082 in the H12 group; c_Kiritimatiellae, o_WCHB1_41, and o_Oscillospirales in the H24 group; f_Muribaculaceae, f_Streptococcaceae, g_Streptococcus, s_Streptococcus_equinus SN033, and o_Lactobacillales in the H36 group; o_Veillonellales Selenomonadales and f_Selenomonadaceae in the H48 group.

### 3.7. Predicted Functions of Ruminal Bacterial Microbiota

This study identified a total of 31 gene families, with 18 of these families exhibiting relative abundances above 1%, as detailed in Table 6. The gene families associated with cell motility and membrane transport in the H0 group were significantly higher than those in the H12 and H24 groups (*p* < 0.05). Conversely, gene families involved in glycan biosynthesis and metabolism, transcription, and translation showed opposite results (*p* < 0.05). The gene families associated with the metabolism of terpenoids and polyketides in the H12 group were significantly higher than those in the H48 group, but this trend was reversed for carbohydrate metabolism (*p* < 0.05). The H24 group had significantly higher gene families involved in amino acid metabolism and replication and repair compared to the H48 group (*p* < 0.05).

## 4. Discussion

### 4.1. Effects of Mixed Inoculum Storage Time on Gas Production and Rumen Fermentation Characteristics

When stored at 39 °C, the residual feed substrates within the rumen fluid, including polysaccharides and proteins, are broken down by microorganisms, resulting in the production of VFA, amino acids, and other byproducts [19]. Our analysis of the mixed inoculum at various storage times prior to in vitro fermentation (as shown in Table 1) demonstrated that levels of NH_3_-N and VFA increased over time, peaking at 36 h, and then decreased after 48 h. This suggests that the feed residues in the rumen fluid were largely consumed within the initial 36 h period under the proper incubation temperature of 39 °C. Given the identical fermentation substrate and a consistent duration of in vitro rumen fermentation, the physicochemical attributes of the mixed inoculum prior to fermentation are influential in shaping the post-fermentation rumen characteristics, as well as gas production. In this study, following a 48 h in vitro fermentation period, the gas production and rumen fermentation characteristics progressively increased until the 24 h of storage, with the exception of MCP and pH levels, after which they began to decline. The turning point for the fermentation characteristics of the mixed inoculum occurs at 36 h, while the post-fermentation characteristics peak at 24 h. This difference can be explained by the fact that fermentation products are determined by a combination of the fermentation substrate, inoculum characteristics, and microbial activity [22]. Most likely, the microbes in the mixed inoculum become less active after 24 h of storage, leading to a numerical decrease in the diversity of rumen microbes after this storage time point. This explanation can also be inferred from the numerically higher microbial diversity observed in the H12 and H24 groups compared to the H36 and H48 groups. Interestingly, the microbial diversity in the H12 and H24 groups was higher than that in the H0 group. Even though the time from collecting rumen contents to the preparation of the mixed inoculum was controlled within an hour, as suggested by Yáñez-Ruiz et al. [7], and the preservation conditions were anaerobic and at 39 °C during this period, it may still trigger stress responses in rumen bacteria. This stress may decrease the diversity of rumen bacteria, which was reflected in the H0 group. As the storage time extended, microbes may employ community defense mechanisms, such as quorum sensing, to cope with stress or adversity [26], which was reflected in the higher diversity observed in the H12 and H24 groups. This also elucidated the reason behind the higher gas production observed in the H12 and H24 groups compared to the H0 group.

Ruminal MCP, often quantified as a crucial source of amino acids for ruminants, is influenced by dietary intake of nitrogen and carbohydrates, as well as by the composition and activity of the microbial population [27]. The elevated MCP levels observed in the H0 group can likely be attributed to the optimal carbon-to-nitrogen ratio present in the mixed inoculum at that time, a balance achieved through a combination of feed residues and fermentation substrates. This balanced ratio is conducive to microbial protein synthesis, as an ideal energy-to-nitrogen ratio is known to enhance the efficiency of protein synthesis by rumen microorganisms [28]. This explanation is indirectly supported by the lower acetate-to-propionate ratio observed in the H0 group, as Lin et al. [29] have indicated that a lower ratio is associated with higher feed utilization efficiency. It is easy to anticipate a lower pH value in the H24 group because the increased production of VFA tends to decrease the pH value, even with the rise in NH_3_-N concentration.

Taking into account the observed decrease in total gas production, ammonia nitrogen, and volatile fatty acids concentrations following a 24 h storage period for the mixed inoculum, it is advised to use the mixed inoculum within this 24 h window. This recommendation is particularly important when assessing gas production and rumen fermentation characteristics in in vitro rumen fermentation tests.

### 4.2. Effects of Mixed Inoculum Storage Time on Rumen Bacterial Diversity and Community Composition

Microbial alpha diversity assesses both the richness and evenness of species, focusing on individuals or specific populations [30]. In this study, richness and evenness both increased before the 24 h storage mark and then declined, with a particularly significant reduction observed after 48 h. The variations in rumen microbial alpha diversity parallel the changes in rumen fermentation characteristics, highlighting the crucial role of rumen microorganisms in the fermentation process. Taxonomic annotation offers a unique perspective on the dynamics of microbial communities in the in vitro fermentation products from the mixed inoculum across different storage times. In this study, Bacteroidota and Firmicutes, the two most prevalent phyla in rumen fluid, accounted for 45.27% and 33.93% of the sample, respectively. Notably, after 12 h of storage, a significant divergence in their proportions was observed, indicating that the storage time of the mixed inoculum greatly influences the composition of the rumen microbiota in in vitro fermentation products. This influence was mirrored in the less prevalent phyla, including Proteobacteria, Verrucomicrobiota, Fusobacteriota, Spirochaetota, Euryarchaeota, and Patescibacteria, with notable deviations from the H0 group becoming apparent after 24 h of storage. Desulfobacterota, encompassing a rich diversity of sulfate-reducing bacteria, is capable of performing sulfate reduction, thiosulfate reduction, sulfur cycling, and other metabolic processes, showcasing strong adaptability and metabolic diversity in harsh environments [31]. In the current study, a significant increase in the abundance of this phylum was observed after the mixed inoculum was stored for 12 h. This increase may be attributed to the ex vivo environment and the introduction of artificial culture media, which likely stimulated Desulfobacterota’s metabolic adaptability to respond to adverse conditions. Ruminobacter, a prominent member of the Succinivibrionaceae family, is a ubiquitous microorganism in the rumens of ruminants, renowned for its sensitivity to environmental changes and its role as a typical starch-degrader [32]. Our study detected a decrease in its relative abundance in the H12 group, contrasting with an increase observed in the H48 group. The initial decline may be due to the swift consumption of easily digestible substances like starch in the initial mixed inoculum, leading to a reduction in the substrate’s starch content. In contrast, the later increase implies that Ruminobacter has adeptly adapted to the changing environmental conditions.

Succiniclasticum, Succinivibrio, and Succinivibrionaceae UCG-002, all belonging to the same family as Ruminobactercan, can convert lactate and succinate into propionate [33]. Notably, the relative abundance of these genera significantly declines with extended storage of the mixed inoculum. This decrease may be due to the fact that the products of rapidly degradable substances in the substrate are mostly propionate, and as the storage time increases, there are fewer of these easily fermentable materials in the substrate. The pattern of change observed in the aforementioned genera corresponds with the fluctuations in both the concentration and proportion of propionate in the rumen fluid. Butyrivibrio and Pseudobutyrivibrio, occupying similar ecological niches within the rumen microbiome, engage in the decomposition of carbohydrates, particularly plant fibers. They break down complex polysaccharides into simpler monosaccharides, which are then fermented to produce short-chain fatty acids like butyrate [34]. Interestingly, the relative abundance of these genera tends to decrease over extended periods of storage, suggesting a potentially limited adaptability to changing environmental conditions. The Lachnospiraceae FCS020 group, Lachnospiraceae AC2044 group, Anaerovibrio, Bacillus, and Probable genus 10 species are capable of fermenting polysaccharides from plant cell walls into simple sugars or short-chain fatty acids like acetate and butyrate, effectively degrading and utilizing fibrous materials [35,36,37]. Our study found that the relative abundance of these groups increased over time with the extended storage of the mixed inoculum, possibly because fiber is not quickly and easily metabolized in the rumen. The Rikenellaceae RC9 gut group and Christensenellaceae R-7 group are both capable of generating short-chain fatty acids that supply energy to rumen epithelial cells, promote cellular repair and regeneration, and contribute to maintaining intestinal health and enhancing nutrient absorption and digestion in animals [38]. Our research observed that the relative abundance of these two groups initially increased before declining after 24 h of mixed inoculum storage, a pattern that aligns with fluctuations in the total VFA concentration, rumen microbial diversity, and in vitro gas production. These findings further substantiate the significant role of these two bacterial groups in enhancing rumen health and overall production efficiency.

LEfSe analysis provided a more in-depth and comprehensive understanding of the variations in microbial populations within the mixed inoculum over different storage times. Negativicutes and Selenomonas facilitate the breakdown of starch and hexoses through key proteins and enzymes [39], explaining their relatively high prevalence in the H0 group. This prevalence is explained by the tendency of microorganisms to prioritize the degradation of readily fermentable materials, such as starch, that are present in the substrate. Acidaminococcales and Acidaminococcaceae are involved in pyruvate metabolic pathways, a key intermediate that can be converted into lactate or succinate, and ultimately into propionate [37]. The high relative abundance of these two microbial groups, along with Clostridia, known as propionate-producing bacteria, in the H0 group correlates with the levels of propionate found in the rumen fluid. Fonseca et al. [40] found an increase in the abundance of Oribaculum within high-starch total mixed ration treatments, highlighting its significant role in starch degradation. High-starch diets activate the propionate pathway by utilizing lactate as an intermediate, thereby promoting the generation of propionate [32]. This finding offers a compelling explanation for the elevated presence of Oribaculum in the H0 group. F082, a newly identified dominant bacterial group in the rumen microbiota with a relative abundance exceeding 1%, exhibits a negative correlation with rumen pH and is notably abundant under high-starch diet conditions [41]. This pattern is consistent with the observed decrease in pH and increase in VFA production following a 12 h incubation of the mixed inoculum in this study. Consequently, it is not surprising to find a higher abundance of F082 in the H12 group.

Kiritimatiellae, Oscillospirales, and WCHB1_41 play vital roles in the degradation and metabolism of the most abundant and recalcitrant sugar polymers in plant cell walls—cellulose and hemicellulose [33]. Their elevated abundance in the H24 group suggests that the mixed inoculum primarily degrades fiber after 24 h of storage, aligning with the observed reduction in VFA production at that time. Muribaculaceae and Lactobacillales engage in cross-feeding interactions and play a crucial role in the degradation of complex polysaccharides, particularly exogenous ones [42]. Streptococcaceae, acting as probiotics, facilitate the hydrolysis and enzymatic decomposition of cellulose [43]. The elevated presence of these microbes in the H36 group suggests that the mixed inoculum remains focused on fiber degradation after 36 h of incubation, though with a different species composition since the 24 h mark. This variation may be due to the dynamic and spatially distributed division of labor among rumen microbes within the complex ecosystem. Veillonellales Selenomonadales and Selenomonadaceae play important roles in energy metabolism and nitrogen utilization under extreme conditions characterized by scarce nutrients, thereby improving the organism’s nutrient utilization efficiency and adaptation to challenging environmental conditions [44]. The high relative abundance observed in this study after 48 h of storage for the mixed inoculum suggests nutrient depletion within the sample. This observation indirectly validates the rationality of the storage duration established for this experiment. The predicted metabolic pathways further validate the observed variations in differential microbes across different storage times of the mixed inoculum. At the 24 h mark, there is a significant shift from degrading readily fermentable substances like starch to less digestible components such as fiber. During this period, metabolic activities in amino acid metabolism, replication and repair, glycan biosynthesis and metabolism, translation, and transcription are intensified. In contrast, by the 48 h mark, the depletion of substrates triggers an upregulation in carbohydrate metabolism and membrane transport as the microbial community responds to the challenges of nutrient scarcity.

Given the substantial changes in the diversity and relative abundances of the majority of rumen bacteria within the mixed inoculum after a 24 h storage period, it is recommended to assess microbial diversity and community structure using the mixed inoculum within the first 24 h of storage for in vitro rumen fermentation studies.

It is important to note that the findings from this study indicate a minor variation in the mixed inoculum after a 12 h storage period. This suggests that the optimal storage time point may be closer to the 0 h mark within the initial 0–12 h window. To determine the most effective storage duration, further refinement of the time points within this interval is necessary. Moreover, to minimize experimental discrepancies and enhance the precision of the outcomes, future research should consider increasing both the number of culture batches and the number of replicates within each treatment group.

## 5. Conclusions

In summary, the storage time of the mixed inoculum affected in vitro fermentation characteristics, rumen bacterial diversity, and community composition. Total gas production, ammoniacal nitrogen, and most volatile fatty acids peaked at 24 h of storage. The richness and evenness of rumen bacteria increased with extended storage times before reaching 24 h. The relative abundances of the majority of rumen bacteria within the mixed inoculum underwent a significant shift after 24 h of storage. Given the observed variations in gas production, rumen fermentation characteristics, microbial diversity, and community structure across different storage times, it is recommended to store the mixed inoculum for no more than 24 h in an anaerobic environment at 39 °C for conducting in vitro rumen fermentation tests.

## Figures and Tables

**Table 1:** Fermentation characteristics of the mixed inoculum under different storage times before in vitro fermentation test.

Item	0 h	12 h	24 h	36 h	48 h
pH value	6.78	6.80	6.80	6.81	6.83
Ammoniacal nitrogen, mg/dL	11.66	16.02	21.59	24.02	22.98
Acetate, mmol/L	21.83	28.29	29.90	31.36	29.45
propionate, mmol/L	6.74	8.42	8.87	9.13	7.45
Isobutyrate, mmol/L	0.20	0.29	0.37	0.41	0.45
Butyrate, mmol/L	4.54	6.24	6.54	6.84	6.28
Isovalerate, mmol/L	0.21	0.50	0.79	0.99	0.97
Valerate, mmol/L	0.40	0.69	0.80	0.88	0.97
Total volatile fatty acids, mmol/L	33.92	44.43	47.26	49.61	45.57
Branched-chain volatile fatty acids, mmol/L	0.81	1.48	1.95	2.28	2.39
Acetate-to-propionate ratio	3.24	3.36	3.37	3.44	3.95

**Table 2:** Effects of mixed inoculum storage time on in vitro total gas production and rumen fermentation characteristics.

Item	Storage Time ^1^					SEM ^2^	*p*-Value
	H0	H12	H24	H36	H48		
Total gas production, mL/g	135.56 b	160.10 a	164.70 a	152.65 ab	151.35 ab	4.129	0.002
pH value	7.01 ab	6.93 ab	6.81 b	6.95 ab	7.05 a	0.048	0.021
Ammoniacal nitrogen, mg/dL	19.31 c	36.34 b	41.38 a	35.81 b	35.35 b	0.851	<0.001
Microbial protein, mg/L	177.63 a	79.94 b	83.40 b	110.85 b	91.52 b	7.947	<0.001
Total volatile fatty acids, mmol/L	77.08 c	94.58 a	94.94 a	89.80 ab	79.10 bc	2.899	<0.001
Concentration, mmol/L							
Acetate	44.58 c	57.05 a	59.28 a	54.41 ab	49.81 bc	1.537	<0.001
Propionate	20.84	19.84	18.57	18.51	16.70	0.982	0.083
Isobutyrate	0.50 d	0.93 ab	1.02 a	0.88 bc	0.75 c	0.030	<0.001
Butyrate	8.68 b	12.44 a	11.45 a	11.91 a	8.51 b	0.389	<0.001
Isovalerate	1.09 d	2.32 ab	2.64 a	2.15 bc	1.79 c	0.084	<0.001
Valerate	1.39 b	2.00 a	1.98 a	1.95 a	1.54 b	0.076	<0.001
Branched-chain volatile fatty acids	2.98 b	5.25 a	5.64 a	4.97 a	4.08 b	0.180	<0.001
Proportion, %							
Acetate	57.97 c	60.32 b	62.44 ab	60.60 b	63.09 a	0.527	<0.001
Propionate	26.97 a	20.98 b	19.55 b	20.61 b	20.98 b	0.582	<0.001
Acetate-to-propionate ratio	2.15 b	2.88 a	3.19 a	2.95 a	3.04 a	0.101	<0.001
Isobutyrate	0.64 c	0.98 ab	1.07 a	0.98 ab	0.95 b	0.025	<0.001
Butyrate	11.21 bc	13.15 a	12.06 b	13.26 a	10.77 c	0.215	<0.001
Isovalerate	1.41 c	2.45 b	2.79 a	2.39 b	2.27 b	0.056	<0.001
Valerate	1.79 c	2.11 a	2.09 ab	2.17 a	1.94 b	0.036	<0.001
Branched-chain volatile fatty acids	3.84 c	5.55 ab	5.95 a	5.53 b	5.16 b	0.095	<0.001

Note: ^1^ H0, H12, H24, H36, and H48 indicate the mixed inoculum used in the in vitro fermentation tests was stored for 0 h, 12 h, 24 h, 36 h, and 48 h, respectively. ^2^ SEM, standard error of the mean. The same lowercase letter indicates no significant difference, while different lowercase letters indicate significant differences.

**Table 3:** Effect of mixed inoculum storage time on alpha diversity metrics of rumen bacterial community.

Item	Storage Time ^1^					SEM ^2^	*p*-Value
	H0	H12	H24	H36	H48		
Chao1	984.53	1061.37	1088.51	1004.18	836.84	64.755	0.087
Observed species	982.25	1059.00	1086.00	999.98	831.00	63.646	0.073
PD whole tree	74.68 ab	77.69 a	75.15 ab	72.83 ab	62.58 b	3.278	0.032
Shannon index	8.16 a	8.52 a	8.63 a	8.28 a	7.55 b	0.124	<0.001
Simpson index	0.9898 a	0.9922 a	0.9917 a	0.9862 ab	0.9758 b	0.002	0.001

Note: ^1^ H0, H12, H24, H36, and H48 indicate the mixed inoculum used in the in vitro fermentation tests was stored for 0 h, 12 h, 24 h, 36 h, and 48 h, respectively. ^2^ SEM, standard error of the mean. The same lowercase letter indicates no significant difference, while different lowercase letters indicate significant differences.

**Table 4:** Effect of mixed inoculum storage time on rumen bacterial community composition (%) at the level of phylum (relative abundance > 0.1%).

Phylum Name	Storage Time ^1^					SEM ^2^	*p*-Value
	H0	H12	H24	H36	H48		
Bacteroidota	32.64 c	49.02 a	52.40 a	48.49 a	41.26 b	1.398	<0.001
Firmicutes	42.19 a	30.17 b	30.17 b	32.64 b	36.12 ab	1.461	<0.001
Proteobacteria	20.06 a	13.00 ab	10.65 b	14.42 ab	16.51 ab	1.927	0.036
Verrucomicrobiota	2.44 bc	3.82 ab	4.33 a	1.86 c d	0.33 d	0.435	<0.001
Fusobacteriota	0.003 b	0.035 b	0.015 b	0.053 b	3.97 a	0.564	<0.001
Desulfobacterota	0.36 b	0.84 a	0.80 ab	0.89 a	0.54 ab	0.107	0.014
Spirochaetota	0.28 c	0.33 c	0.54 bc	0.98 ab	0.99 a	0.103	<0.001
Euryarchaeota	1.12 ab	1.78 a	0.21 b	0.09 b	0.04 b	0.288	0.001
Patescibacteria	0.40 b	0.44 ab	0.60 a	0.22 c	0.12 c	0.038	<0.001
Actinobacteriota	0.22	0.13	0.05	0.12	0.01	0.078	0.442

Note: ^1^ H0, H12, H24, H36, and H48 indicate the mixed inoculum used in the in vitro fermentation tests was stored for 0 h, 12 h, 24 h, 36 h, and 48 h, respectively. ^2^ SEM, standard error of the mean. The same lowercase letter indicates no significant difference, while different lowercase letters indicate significant differences.

**Table 5:** Effect of mixed inoculum storage time on rumen bacterial community composition (%) at the level of genus (relative abundance > 1%).

Genus Name	Storage Time ^1^					SEM ^2^	*p*-Value
	H0	H12	H24	H36	H48		
Rikenellaceae RC9 gut group	7.82 c	13.91 b	17.42 a	15.85 ab	14.36 b	0.690	<0.001
Ruminobacter	8.88 ab	5.07 b	7.39 ab	11.35 ab	15.26 a	1.912	0.011
Prevotella	10.49	9.25	9.95	7.85	9.85	1.246	0.632
Succiniclasticum	10.51 a	9.04 a	9.58 a	4.63 b	2.82 b	1.026	<0.001
Anaerovibrio	1.60 b	0.95 b	0.33 b	2.54 b	8.44 a	1.193	0.001
Succinivibrio	4.82 a	5.11 a	1.35 b	2.08 b	0.94 b	0.287	<0.001
Succinivibrionaceae UCG-002	5.47 a	1.88 b	1.43 b	0.18 c	0.11 c	0.272	<0.001
Pseudobutyrivibrio	4.80 a	0.84 b	0.46 b	0.90 b	0.65 b	0.135	<0.001
Butyrivibrio	2.14 a	0.98 c	0.95 c	1.64 b	1.09 c	0.108	<0.001
Lachnospiraceae FCS020 group	0.48 d	0.59 d	1.02 c	1.70 b	2.42 a	0.066	<0.001
Probable genus 10	1.22 b	0.63 c	0.45 c	1.58 a	1.71 a	0.074	<0.001
Bacillus	0.09 b	0.18 b	0.13 b	0.66 b	4.21 a	0.175	<0.001
Christensenellaceae R-7 group	0.83 cd	1.37 ab	1.40 a	1.02 bc	0.52 d	0.088	<0.001
Lachnospiraceae AC2044 group	0.76 b	0.54 b	0.80 b	1.41 a	1.58 a	0.105	<0.001

Note: ^1^ H0, H12, H24, H36, and H48 indicate the mixed inoculum used in the in vitro fermentation tests was stored for 0 h, 12 h, 24 h, 36 h, and 48 h, respectively. ^2^ SEM, standard error of the mean. The same lowercase letter indicates no significant difference, while different lowercase letters indicate significant differences.

**Table 6:** Effect of mixed inoculum storage time on the relative abundance (%) of the predicted metabolic pathways in the rumen bacterial microbiome.

Item	Storage Time ^1^					SEM ^2^	*p*-Value
	H0	H12	H24	H36	H48		
Carbohydrate metabolism	13.85 ab	13.38 c	13.57 bc	13.85 ab	14.10 a	0.098	<0.001
Metabolism of cofactors and vitamins	13.64	13.56	13.72	13.37	13.69	0.120	0.258
Amino acid metabolism	13.03 ab	13.05 ab	13.17 a	13.00 ab	12.92 b	0.053	0.037
Metabolism of terpenoids and polyketides	9.30 ab	9.88 a	9.71 ab	9.65 ab	9.23 b	0.140	0.018
Metabolism of other amino acids	7.07	6.99	6.95	6.84	6.93	0.139	0.850
Replication and repair	6.28 b	6.42 ab	6.48 a	6.38 ab	6.28 b	0.033	0.001
Energy metabolism	5.26	5.49	5.29	5.33	5.16	0.083	0.111
Lipid metabolism	4.89	4.91	4.92	5.10	4.96	0.076	0.357
Glycan biosynthesis and metabolism	4.55 c	4.93 ab	5.08 a	4.85 abc	4.68 bc	0.078	0.001
Translation	3.55 bc	3.65 a	3.65 a	3.57 ab	3.47 c	0.019	<0.001
Folding, sorting and degradation	3.40	3.33	3.35	3.33	3.36	0.035	0.722
Xenobiotics biodegradation and metabolism	2.39	2.50	2.37	2.34	2.31	0.151	0.915
Biosynthesis of other secondary metabolites	2.28	2.33	2.38	2.39	2.28	0.035	0.096
Nucleotide metabolism	2.10	2.14	2.16	2.14	2.12	0.013	0.101
Cell motility	2.32 a	1.64 bc	1.41 c	1.83 b	2.33 a	0.079	<0.001
Membrane transport	1.80 ab	1.57 c	1.58 c	1.71 b	1.83 a	0.023	<0.001
Cell growth and death	1.65	1.63	1.67	1.66	1.66	0.012	0.355
Transcription	0.98 b	1.05 a	1.05 a	1.01 ab	1.00 ab	0.012	0.004

Note: ^1^ H0, H12, H24, H36, and H48 indicate the mixed inoculum used in the in vitro fermentation tests was stored for 0 h, 12 h, 24 h, 36 h, and 48 h, respectively. ^2^ SEM, standard error of the mean. The same lowercase letter indicates no significant difference, while different lowercase letters indicate significant differences.

## Data Availability

The raw sequencing data were deposited in the NCBI’s Sequence Read Archive (SRA) database with the accession number PRJNA1185238.
